# Physicochemical characterization and rheological properties of magnetic elastomers containing different shapes of corroded carbonyl iron particles

**DOI:** 10.1038/s41598-020-80539-z

**Published:** 2021-01-13

**Authors:** Nurul Liyana Burhannuddin, Nur Azmah Nordin, Saiful Amri Mazlan, Siti Aishah Abdul Aziz, Noriyuki Kuwano, Siti Khumaira Mohd Jamari, Ubaidillah

**Affiliations:** 1 Engineering Materials and Structures (eMast) iKohza, Malaysia-Japan International Institute of Technology (MJIIT), Universiti Teknologi Malaysia, Jalan Sultan Yahya Petra, 54100 Kuala Lumpur, Malaysia; 2 International Center, 1 Chrome-28-1 Tamazutmi, Setagaya, Tokyo 158-0087 Japan; 3 The Ultramicroscopy Research Center, Kyushu University, 744 Motooka, Nishu-ku, Fukuoka, 819-0395 Japan; 4 Department of Mechanical Engineering, Faculty of Engineering, Universitas Sebelas Maret, J1. Ir. Sutami 36A, Kentingan, Surakarta, Central Java 57126 Indonesia

**Keywords:** Mechanical engineering, Soft materials, Structural materials

## Abstract

Carbonyl iron particles (CIPs) is one of the key components in magnetic rubber, known as magnetorheological elastomer (MRE). Apart from the influence of their sizes and concentrations, the role of the particle’ shape is pronounced worthy of the attention for the MRE performance. However, the usage of CIPs in MRE during long-term applications may lead to corrosion effects on the embedded CIPs, which significantly affects the performance of devices or systems utilizing MRE. Hence, the distinctions between the two types of MRE embedded in different shapes of spherical and plate-like CIPs, at both conditions of non-corroded and corroded CIPs were investigated in terms of the field-dependent rheological properties of MRE. The plate-like shape was produced from spherical CIPs through a milling process using a rotary ball mill. Then, both shapes of CIPs individually subjected to an accelerated corrosion test in diluted hydrochloric (HCl) at different concentrations, particularly at 0.5, 1.0, and 1.5 vol.% for 30 min of immersion time. Eight samples of CIPs, including non-corroded for both CIPs shapes, were characterized in terms of a morphological study by field emission scanning electron microscope (FESEM) and magnetic properties via vibrating sample magnetometer (VSM). The field-dependent rheological properties of MREs were analyzed the change in the dynamic modulus behavior of MREs via rheometer. From the application perspective, this finding may be useful for the system to be considered that provide an idea to prolong the performance MRE by utilizing the different shapes of CIPs even when the material is fading.

## Introduction

Carbonyl iron particles (CIPs) have been widely used in magnetic materials, known as magnetorheological (MR) due to their high permeability, high magnetic saturation and low remnant magnetization^1^. MRE is a magnetic rubber material, composed of CIPs that are interlocked in the elastomeric matrix. Due to its extensive applications in vibration and shock absorption, noise reduction, and other areas, MRE has gained broader attention for various devices such as adaptively tuned vibration absorbers, mass dampers, sensors, actuators, and base isolator as semi-active control devices^2–4^. In the past few years, more attention has been paid to enhance the magnetic effect of MRE through the interparticle interaction between magnetic particles and solid-like matrix. It was reported that the properties of MRE could be improved by controlling the composition and particle size of CIPs^5,6^. Apart from these two factors, different shapes of CIPs and their influence on the resultant rheological properties of MRE would also be considered. Accordingly, a considerable amount of literature that exist are related to the influence of CIPs in different shapes, such as spherical^7–10^, irregular^11,12^, rod^13^, and flaky or plate-like^14–16^, in which those studies have shown a tremendous effect on the performance of MRE. The CIPs with spherical-shaped particles are the most frequently used in MR materials^17^ due to their soft magnetic characteristics, relatively smooth surface, ease of reproducibility and higher mechanical stability. Ubaidillah et al.^18^ have provided a statistics on the distribution diagram of magnetic particles and it has been noted that most researchers have preferably used the spherical shape of particles in the preparation of MRE. Besides, the past few decades have shown a rapid development in the field of MRE that used spherical CIPs as a candidate of magnetic particles in the elastomeric matrix^19–21^.

It is known that different particle shapes play an essential role in the resultant mechanical or rheological properties of MRE. One of the critical aspects in obtaining enhanced properties of MRE is having a higher aspect ratio (length to width) of CIPs, such as flaky or plate-like shapes that are embedded in the elastomeric matrix. Briefly, under the influence of the magnetic field, the CIPs would begin to vibrate and produce magnetic moments between the particles, which would then tend to form a chain-like structure. A smaller gap exhibited between the plate-like particles in the elastomeric matrix will result in the improvement of the interaction between the CIPs and polymer matrix, which leads to the enhancement of the respective rheological response (stiffness) of the MRE. A recent study by Hapipi et al.^22^ demonstrated the excellent rheological performance of MRE that contained the plate-like shape of CIPs. They claimed that MREs having a plate-like CIPs, particularly in anisotropic condition possessed an improvement in terms of storage modulus and loss modulus compared with those MRE reinforced with spherical CIPs. Meanwhile, Zheng et al.^23^ concluded that the aligned flaky or plate-like CIPs in a silicone rubber-based matrix has enhanced the MR effect by about 39.2% with a higher initial storage modulus as compared to the case of isotropic CIPs in the MRE. This result was attributed to a greater interaction between the flaky or plate-like CIPs with the elastomeric matrix.

On the other hand, some other studies have shown the use of the plate-like shape of CIPs in other MR materials that improved the resultant properties of the materials. For instance, it was reported that the issue of sedimentation in magnetorheological fluid (MRF)^24^ was remarkably reduced when the spherical shape of CIPs was replaced by the plate-like (or flakes) shape, and in return, the field-dependent rheological properties of MRF were enhanced. Upadhyay et al.^25^ found that a flake-based MRF improved the storage modulus and MR effect of MRF compared to the spherical-based MRF. Besides, the sedimentation rate of the particles was decreased by about 50%. Shilan et al.^26^ showed that the sedimentation rate was reduced by 20% upon using the plate-like CIPs, due to an increased in the friction force between the particles. They reported that the saturation magnetization of MRF with plate-like CIPs was higher around 8%, and the yield stress value increased up to 270% in comparison to the spherical CIPs-based MRF. In another study by Mohamad et al.^27^, they conducted a comparative work on the plate-like CIPs over the spherical shaped in magnetorheological grease (MRG). The results showed that under the influence of the magnetic field, a distinct chain-like structure of plate-shaped particles was formed, causing in a higher value of the storage modulus of MRG. Overall, these outcomes suggested that a higher aspect ratio and a broader surface area of the plate-like particles resulted in an improvement in the performance of MR materials due to more extensive interactions between the CIPs under the applied magnetic field. Besides, the practicability and easiness of producing plate-like CIPs from the spherical shape in the one-step process have already been proven^28,29^. Therefore, all these findings have contributed to the knowledge concerning the significance of utilizing plate-like CIPs in MR materials.

However, despite the advantages that have enhanced the performance of MR materials, there is an increasing concern over using the plate-like shape of CIPs, as well as spherical or other shapes of the particles. Although the CIPs embedded in an elastomeric medium are not directly exposed to the external environment, the air and moisture, even so, they can diffuse into the MR medium and act as corrosion agents that could affect the magnetic properties of the CIPs. This phenomenon in return would affect the inter-particle bonding between the CIPs and the elastomeric matrix^30,31^ that subsequently deteriorate the properties and performance of the MRE. It is believed that the oxidized layer might be formed on the surface of CIPs as a result of the corrosion process-induced to the metal iron of CIPs which may affect its respective magnetic properties and the MRE.

In an investigation of corroded particles in MRE, Aziz et al.^32^ reported a reduction of MR effect up to 114% for MRE with corroded spherical CIPs. However, they claimed that the MRE containing corroded spherical CIPs exhibited a small increase in the properties respective to the storage modulus under both off- and on-state conditions. It was suggested due to the existing outer layer of the corroded CIPs that acted as a bridge to strengthen the bonding between the CIPs, promoting the agglomeration of particles that occurred during the corrosion process which resulted in the increase of interactions between interparticle of the CIPs themselves. This finding is also susceptible to being inconsistent and unable to demonstrate the overall performance of MRE, due to the agglomeration of corroded CIPs that have been embedded in the elastomeric matrix. In contrast, a study done by Sedlacik et al.^30^ focused on resolving the oxide layer that formed on the CIPs that has deteriorated the properties of MRE, via modification on the surface particles. The modification was believed could control the corrosion phenomenon towards the CIPs and improve the compatibility between the magnetic particles and the matrix phase. Thus, it would delay the degradation of the MRE upon a long-term use of the material. As the formation of an oxide layer on the magnetic particles has weakened the properties of the MRE, it is plausible that the plate-like CIPs might experience a greater corrosion phenomenon on its surface due to the greater aspect ratio and surface area (larger diameter and thin thickness). Therefore, the magnetic behavior of MRE with plate-like CIPs, as well as the rheological properties of MRE would be degraded more than that of MRE with spherical CIPs after a prolonged use although the plate-like based MRE has advantages in enhanced properties.

Nevertheless, there is much uncertainty about the formation of those layers on the surface of both CIPs shapes, especially towards the performance of MRE after long-term usage. In addition, the number of studies related to the influence of corrosion phenomena on the properties of MRE is still somewhat limited. Therefore, this current study was carried out to clarify the influence of corrosion process on different shapes of CIPs and the subsequent effects on the rheological behavior of MRE. To the best of the authors’ knowledge, there is no comparative analysis of the difference between corroded CIP shapes in MRE materials. In order to achieve this goal, different shapes of CIPs, particularly spherical and plate-like, would undergo an accelerated corrosion process in various concentrations of HCl (0.5, 1.0, and 1.5 wt%) and a comparative work towards the magnetic properties of MREs were investigated. The test will be done under the dynamic (oscillatory) behaviors related to set strains, frequencies, and magnetic fields, at off- and on-state conditions that would affect the characterization of field-dependent materials and rheological properties of MRE. This kind of study is closely related to how the shape of corroded particles would play a significant influence on the MRE and the findings would be an indicator to control the MRE performance for long-term operation in the application of MR devices or systems.

## Experimental methods

### Raw materials

Carbonyl iron particles (CIPs) of a spherical shape with the range size from 3 to 5 µm in diameter was used as the magnetizable particles from CK Materials Lab. Hydrochloric acid (HCl, Riendemann Schmidt Chemical, Grade AR, 37%) was selected as a medium for the accelerated corrosion test. The silicone rubber (Nippon Steel, Model NS 625A) was used as a solid matrix of elastomer together with the curing agent (Nippon Steel, Model NS 625A) in the liquid state.

### Preparation of plate-like CIPs

The CIPs of plate-like shape were produced from the spherical ones with a rotary ball mill from Tencan Company (Model: QM-5) as follows. The spherical CIPs were milled with zirconia balls of a diameter of 5 mm in an alumina milling pot used as the grinding medium of the milling process for 40 h with a ball-to-powder ratio of 20:1. During the milling process, pure ethanol was added as a process controlling agent (PCA) to avoid the adhesion problem and improve the efficiency of obtaining the plate-like shape of CIPs^26^.

### Accelerated corrosion test

In order to accelerate the corrosion process, the accelerated corrosion test was applied by immersion in diluted hydrochloric acid (HCl). 0.5 vol.% of HCl in 100 ml of distilled water. Then, 30 g from each shape of the CIPs were slowly poured into the diluted HCl and the solution was stirred for one minute continuously, and the mixture was left for 30 min. After that, the corroded CIPs were taken out from the HCl solution using a magnetic bar and were washed with ethanol several times to eliminate the diluted residues. Afterward, the corroded CIPs were subsequently undergoing a drying process, which involved in three-stages. At first, a sonication or ultrasonic processing was performed to avoid re-agglomeration of the corroded particles. Next, the solution was ready for filtration by using the vacuum pump and, lastly, followed by the sieving process in getting the uniform particle’s size. The same treatment of accelerated corrosion test was carried out with 1.0 and 1.5 vol.% of HCl to study the effect of different corrosion rates on the rheological behavior of MREs containing different shapes of corroded CIPs.

### Particles characterization

The surface morphology of non-corroded and corroded CIPs for both spherical and plate-like shapes were observed with a field emission scanning electron microscope (FESEM), Model: JSM-7800F of Microscopy Laboratory MJIIT, UTM, that equipped with energy-dispersive X-ray spectroscopy (EDS) for the elemental compositions. The test was conducted in a high vacuum mode at an acceleration voltage of 2 kV and a magnification of 16,000x. Through the observation via FESEM, the surface morphology or tomography of the CIPs for both shapes before and after the corrosion process were obtained. Besides, a vibrating sample magnetometer (VSM) (Lakeshore, USA Model: 7404 Series) was used to analyze the magnetic properties of the non-corroded and corroded CIPs samples that underwent different corrosion rates, at ambient temperature. All samples were subjected to measurement of respective saturation magnetization under an applied magnetic field of ± 15 kOe.

### Fabrication of MREs

40 wt% of liquid silicone rubber and 60 wt% of CIPs were mixed at room temperature using a mechanical stirrer at 280 rpm for 10 min. Subsequently, the mixture was then poured into a steel mold and cured under the absence of the magnetic field. In such a way, the MRE was cured in isotropic curing condition. Then, the fabrication steps were repeated for MREs containing various corrosion rates of CIPs, after being immersed in 0.5, 1.0, and 1.5 vol.% HCl, respectively for both shapes. In total, eight types of MREs including two MREs containing non-corroded of spherical (MRE-S NC) and plate-like (MRE-P NC) CIPs for comparison purposes, and six MREs containing corroded CIPs for both shapes (MRE-S: MRE with spherical CIPs; MRE-P: MRE with plate-like CIPs) with different corrosion rates were individually fabricated. The prepared MREs were tabulated in Table 1.

**Table 1:** Compositions of MREs.

Name of sample	Concentration of (HCl) for corrosion test (vol.%)	Spherical CIPs (wt%)	Plate-like CIPs (wt%)	Silicone rubber (wt%)
MRE-S NC	–	60	–	40
MRE-S 0.5	0.5	60	–	40
MRE-S 1.0	1.0	60	–	40
MRE-S 1.5	1.5	60	–	40
MRE-P NC	–	–	60	40
MRE-P 0.5	0.5	–	60	40
MRE-P 1.0	1.0	–	60	40
MRE-P 1.5	1.5	–	60	40

### Rheological test of MRE

The rheological test of MREs under various conditions was performed using an oscillatory shear-mode type of rheometer (MCR 302, Anton Paar Company, Germany) equipped with a controllable magnetic field, supported by an MR device (MRD70/1 T) and profiled parallel plate measuring system (PP20/MRD/T1/P2). A parallel plate measuring system (PP20) with a diameter of 20 mm was used at a gap of 1 mm in thickness. The magnetic flux density, B, could be changed from 0 to approximately 650 mT by varying the driving currents for an electromagnet from 0 until 4 Amps. All tests of strain sweep, frequency sweep and magnetic field sweep test were carried out at room temperature. In order to get a linear viscoelastic region (LVE) of MREs, the strain sweep of 0.001–10% was performed at a constant frequency of 1 Hz. The frequency sweep test was then performed between 0.1 to 50 Hz with the adopted strain in the LVE to obtain the storage modulus as a function of frequency. Afterward, the magnetic field sweep test was carried out to measure the MR effect of MRE, by varying the applied currents from 0 to 4 Amps under the fixed applied strain and frequency, and the effect of corrosion condition and shapes of CIPs were analyzed.

## Experimental results and discussion

### Morphological characterization of CIPs

**Figure 1:**
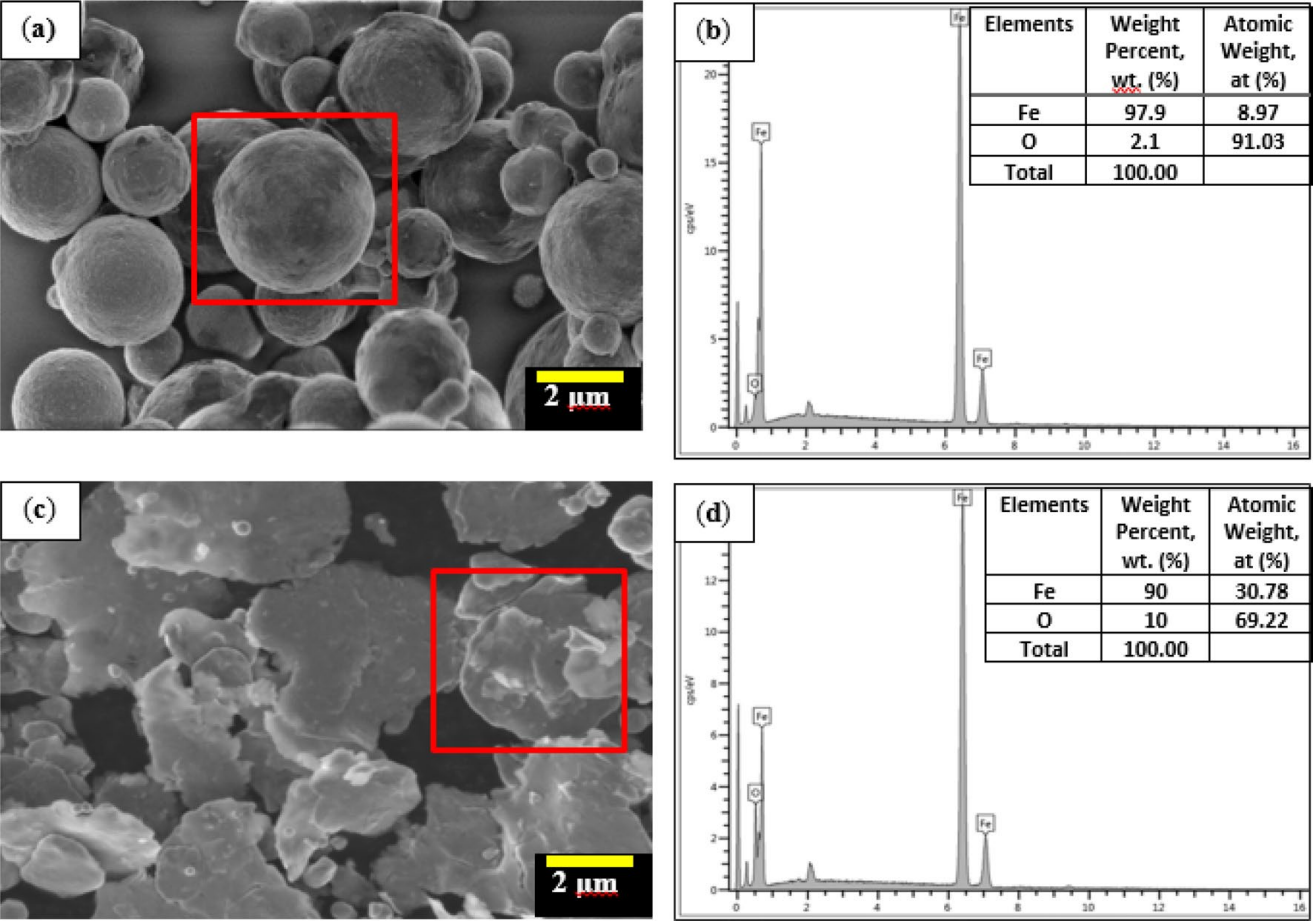
FESEM micrograph of non-corroded CIPs at different particle shapes; (**a**) spherical (before milling) and (**b**) EDX analysis for spherical shape, and (**c**) plate-like (after milling) and (**d**) EDX analysis for plate-like shape.

Error! Reference source not found. shows the FESEM micrograph of the CIPs before and after the milling process. As shown in Fig. 1a, the original unmilled CIPs were spherical and had smooth roundness; the average sizes ranged between 2 and 5 μm. The morphology of the milled CIPs, after the spherical CIPs underwent 40 h of milling process, transformed into a flattened shape of the particles known as plate-like and it is presented in Fig. 1b. It is evident from the obtained images that the milling process could alter the non-corroded CIPs from spherical to plate-like shape. The average aspect ratio of the plate-like CIPs as observed was notable compared to the particle size of spherical CIPs. This present finding seemed to be consistent with other researchers^25,33^, who reported that the plate-like CIPs were observed to be flattened into various sizes, following the range sizes of the spherical CIPs that underwent ball milling process. It can, therefore, be assumed that the thickness of the plate-like CIPs also decreased in correspondence to the broader diameter of the flattened CIPs.

**Figure 2:**
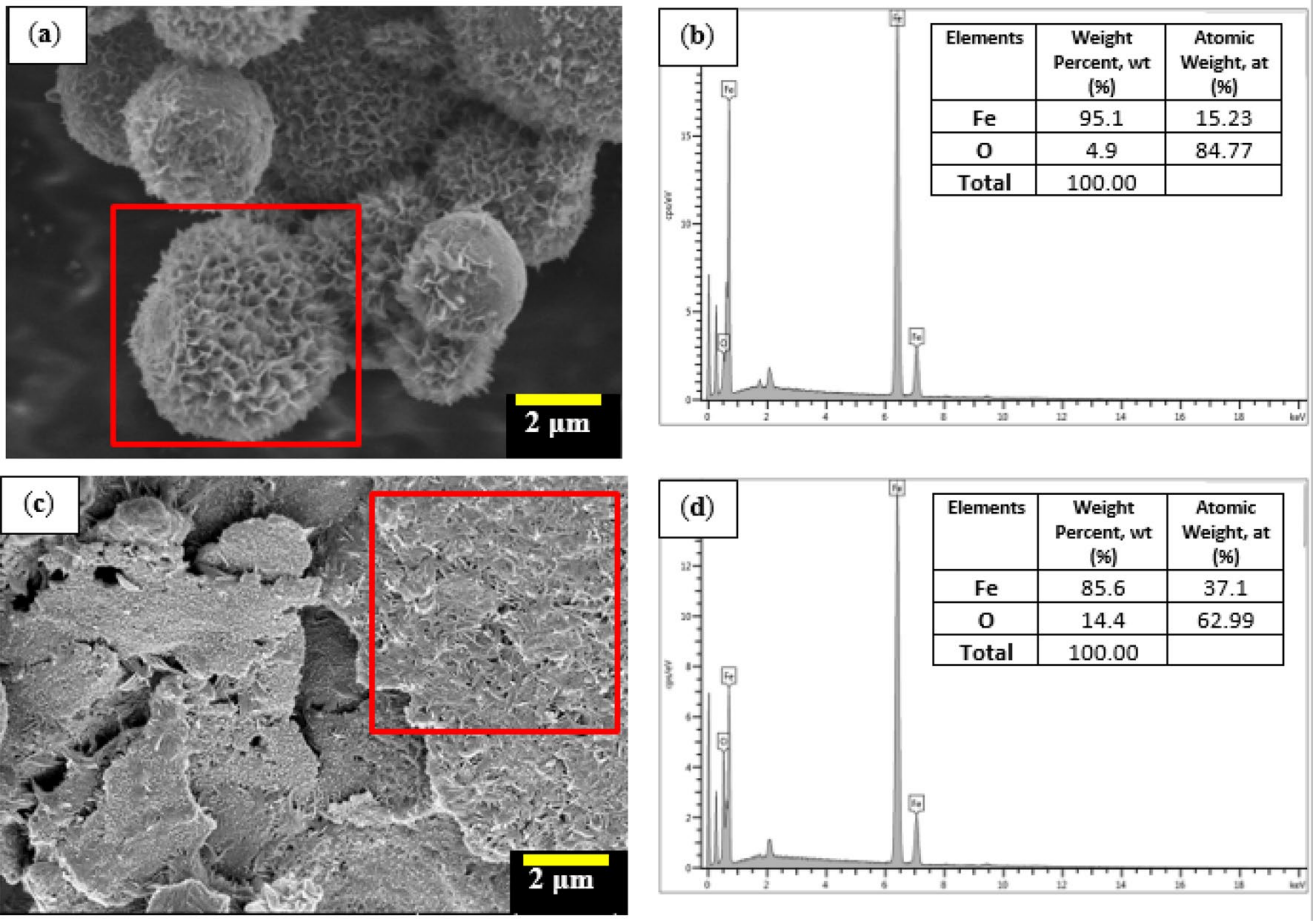
FESEM micrograph for (**a**) corroded spherical CIPs and (**b**) EDS analysis for corroded spherical CIPs at selected points, and (**c**) corroded plate-like CIPs and (**d**) EDS analysis for corroded plate-like CIPs at selected points.

Meanwhile, the surface morphology of spherical and plate-like CIPs that underwent the corrosion process is shown in Fig. 2. It has been observed that both morphologies of the CIPs experienced changes by forming an unknown structure which was white in color on the surface of the CIPs. Indeed, the existence of the non-uniformed and sharp-edged structure, looked like a fluffy ice crystal, has been crystallized on the surface of the spherical CIPs as observed in Fig. 2a. Meanwhile, the coarse and white-dotted structures were observed on the surface of the plate-like CIPs as in Fig. 2c. This result indicates that the surface of CIPs in both shapes becomes rougher as compared to the condition in Fig. 1. The formation of rough surfaces on both CIPs is believed to be due to a reaction occurred during the immersion of CIPs in the diluted 1.0 vol.% HCl. When the iron was exposed to diluted HCl, the chemical reaction caused a formation of ferrous chloride, FeCl_2_, on the surface of CIPs that would further react with diffused air to form a solid oxide layer on its surface. The CIPs were said to undergo an oxidation process. This kind of reaction has simultaneously caused the presence of a rougher layer on the surface of particles, as shown in Fig. 2. In order to determine the presence of these oxide layers on the surface of CIPs, EDX point analysis was performed to identify the elements involved on the surface structure of both CIPs shapes. It showed that the increase in percentage of oxygen (wt%) increased corresponded to the formation of oxide layers on its surface as compared to non-corroded condition in the individual shape of CIPs, either circular or plate-like (Fig. 1). It was also noted that the wt% of oxygen was noted higher for plate-like CIPs as compared to a spherical shape, indicating a slightly more oxidation process experienced by the plate-like shape of CIPs. Indeed, the increment of oxygen content was about ~ 3 wt% for circular shape and around 4.4% for the plate-like shape of CIPs. The quantitative data for both corroded shapes of CIPs are illustrated in Fig. 2b,d. These EDX analysis results provided further support to the hypothesis that the CIPs either in spherical or plate-like shapes, which undergo a corrosion process will have higher oxygen (O) content as a result of a chemical reaction with the surrounding.

### Magnetic properties of CIPs

**Figure 3:**
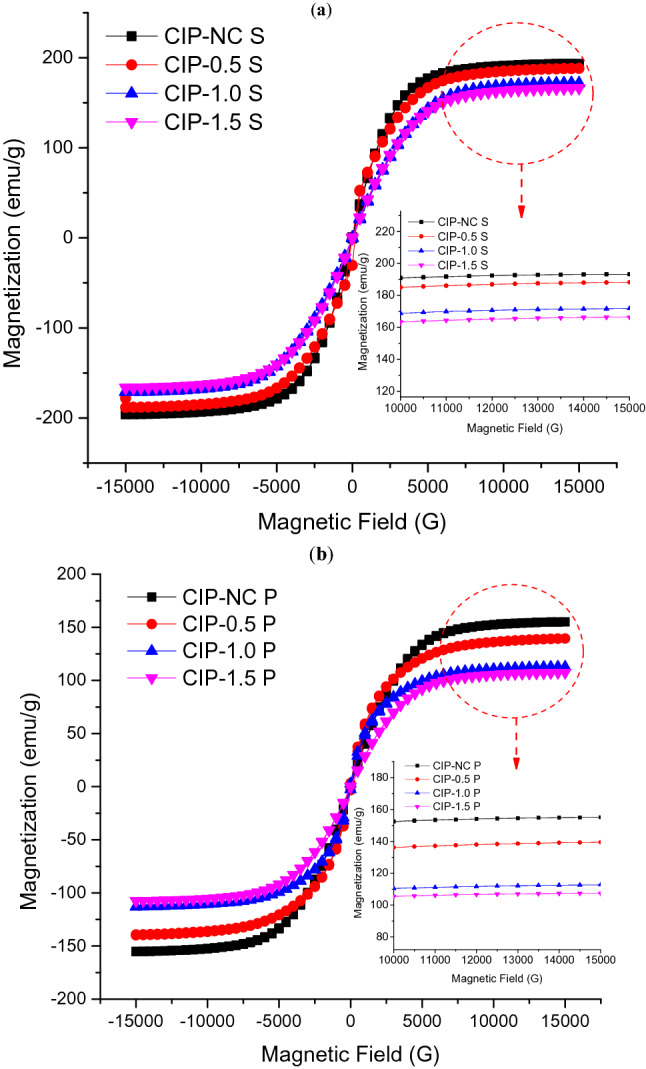
Magnetization curves of different shapes of CIPs (**a**) spherical CIPs and (**b**) plate-like CIPs as a function of magnetic field correspond to various concentrations of diluted HCl.

The change of the surface morphology of CIPs by the corrosion process is believed can influence the magnetic properties, which will degrade the performance of MRE. The magnetic properties of the spherical and plate-like CIPs were measured in a powder state via VSM under the influence of a magnetic field in the range of − 15 to + 15 kOe, at ambient temperature. The magnetization curves of all samples after the corrosion process by immersing in various concentrations of HCl, 0.5, 1.0, and 1.5 vol.% are depicted in Fig. 3. The result showed that the non-corroded spherical CIPs have the highest magnetization saturation value, M_s_ at about 196.05 emu/g, compared to the corroded spherical CIPs samples. The M_s_ value of the non-corroded spherical CIPs, however, decreased from 196.05 to 178.30, 171.20, and 166.50 emu/g, respectively to the concentration of the corrosive solution, 0.5, 1.0, and 1.5 vol.% HCl. This indicated that the decrement in M_s_ value was in accordance with the concentration of HCl, as shown in Fig. 3a. However, as displayed in Fig. 3b, the M_s_ value of the non-corroded plate-like CIPs exhibited around 155.30 emu/g, which was lower than that of the non-corroded spherical CIPs. The M_s_ value was decreased as the spherical CIPs was changed to the plate-like shape. It was believed to be due to the oxidation induced on the surface of the plate-like CIPs during the milling process. This result is correlated with the finding in Fig. 1d that shows the reading of oxygen content (wt%) for the non-corroded plate-like CIPs was higher as compared to non-corroded circular CIPs Fig. 1c. The outcome is in a good agreement with those of other studies^14,27^. On the other hand, the M_s_ value of the corroded plate-like CIPs showed the same trend as in corroded spherical shape, which was remarkably reduced from 155.30 emu/g of non-corroded condition to 140.0, 113.1 and 107.5 emu/g for 0.5, 1.0 and 1.5 vol.% HCl of the corrosion process, respectively.

In brief, all corroded CIPs in both shapes exhibited lower magnetization values, M_s_ attributed to the existence of oxide layers on the surface of the CIPs. This phenomenon can weaken the magnetic interactions between the CIPs, thus reducing the magnetic moments among particles and towards the applied magnetic field. As the concentration of HCl increased, the M_s_ value showed a further decreasing trend, accordingly, exhibiting more corrosion process experienced by the CIPs. In addition, the M_s_ values of the corroded plate-like CIPs were much lower than those of corroded spherical CIPs in all corrosion conditions, suggesting that the larger surface area of the plate-like CIP resulted in higher exposure to the corrosive environment.

A summary of the magnetic saturation, M_s_ and coercivity, H_c_ for both CIPs shapes that were extracted from Fig. 3 is tabulated in Table 2. As the hysteresis loops were very narrow, a remanence, H_r_ for both CIPs was negligible. Table 2 also illustrates the reduction in M_s_ values (%) for both CIPs shapes was normalized to the non-corrosion condition. It was noted that the reduction of the M_s_ value was about 27% for the plate-like shape, larger than spherical-shape of CIPs around 13%, in 1.0 vol.% HCl. Greater oxide layers might be formed on the surface of plate-like CIPs due to having the higher aspect ratio and surface area, subsequently weakened the interaction of magnetic forces between the particles as well as towards the applied magnetic field, resulting in lower M_s_ values.

**Table 2:** Comparison of magnetic properties between different shapes of CIPs.

CIPs samples	Magnetization, M_s_ (emu/g)		Percentage Reduction of M_s_ (%)		Coercivity, H_c_ (G)		Remanence, M_r_ (G)	
	Spherical	Plate-like	Spherical	Plate-like	Spherical	Plate-like	Spherical	Plate-like
Non-corroded	196.05	155.30	–	–	9.923	45.61	0.701	0.347
0.5 vol.% HCl	178.30	140.00	9.05	9.85	9.844	42.82	0.439	0.331
1.0 vol.% HCl	171.20	113.10	12.68	27.17	8.679	41.20	0.452	0.319
1.5 vol.% HCl	166.50	107.50	15.07	30.78	7.189	38.78	0.362	0.268

Besides, it was also observed that the value of coercivity, H_c_ of spherical CIPs has decreased with the increased concentrations of the corrosive environment. Likewise, the plate-like CIPs have a similar trend of H_c_ behavior, in which H_c_ reduced upon exposure to more corrosion condition. However, the H_c_ value of the non-corroded plate-like CIPs was found significantly higher compared to the non-corroded spherical CIPs. It might be due to the change of the CIPs from spherical to the plate-like shape, which offered a high value of aspect ratio that subsequently affected the increment of the coercivity of the CIPs. In addition, the phenomena can also be explained by the fact that a smaller coercivity implied that the spherical CIPs was a softer magnetic material^34^. In contrary, the reduction of H_c_ values that corresponded to the increasing concentration of HCl in both CIPs shapes was due to the decrement in magnetic properties of the CIPs with increment corrosion effects accordingly.

## Results of rheological properties of MREs

### Strain sweep measurement

**Figure 4:**
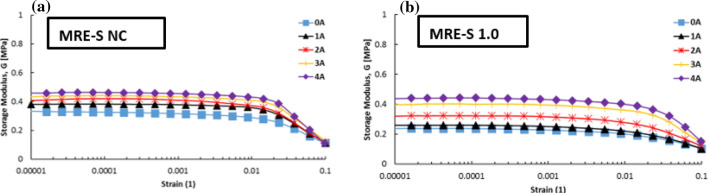
Dependence of storage modulus of MREs towards applied strains containing spherical CIPs with selected corrosion rate (**a**) 0 and (**b**) 1.0 vol.%.

The changes of the storage modulus were measured by the strain sweep test with the applied strain amplitude from 0.001 to 10% under various magnetic field strengths with a constant frequency of 1 Hz. Figure 4 shows the result of measuring the storage modulus of MRE-S samples as a function of applied strains under different applied currents, at selected HCl concentrations, particularly 0.0% and 1.0 vol.%. The figure shows that the storage modulus was constant over a low strain amplitude, particularly up to 0.01% strains for each sample which suggests that the region was the linear viscoelastic region (LVE) of MRE. In this work, the LVE limit is defined as any points within the constant storage modulus, G in which the structure in the MRE would return to its original position after removal of the applied stress.

On the other hand, a sharp decline of the storage modulus, G with further increasing strain amplitudes, was referred to as the non-linear viscoelastic (NLVE) region. Besides, the storage modulus increased with increasing magnetic flux density, which could be attributed to a more robust network structure at a higher magnetic field. Normally, in a reinforced elastomer composite (MRE), two types of interactions would occur, which are particle–matrix and particle–particle interactions. These subsequently increased the storage modulus of the MRE. This phenomenon could be observed for each of the MRE-S samples as presented in Fig. 4a,b, individually. As more magnetic fields were applied, the MRE became stiffer and more stress was needed to overcome the LVE limit.

**Figure 5:**
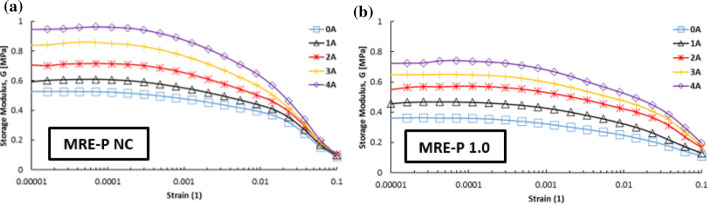
Dependence of storage modulus of MREs towards applied strains containing plate-like CIPs with selected corrosion rate (**a**) 0 and (**b**) 1.0 vol.%

In the same way, the plate-like CIPs in MRE experienced corrosion process upon 1.0 vol.% concentration of HCl. Thus, the storage modulus of the MRE-P samples was slightly reduced with a shorter LVE region as illustrated in Fig. 5b. The important finding was that the LVE region of MRE-P samples (Fig. 5) was shorter than the MRE-S samples (Fig. 4). This phenomenon was observed for MRE either with non-corroded or corroded CIPs. As the plate-like CIPs with higher surface area and aspect ratio embedded in the elastomeric matrix, the storage modulus of the MRE-P samples increased. In fact, this might be due to the stronger particle–particle interactions between the plate-like CIPs and the elastomeric matrix especially towards applied magnetic fields. Presence of corroded layer on the plate-like CIPs, however, has decreased the capability of MRE to respond to applied magnetic fields thus reducing its respective storage modulus, as illustrated in Fig. 5b.

**Figure 6:**
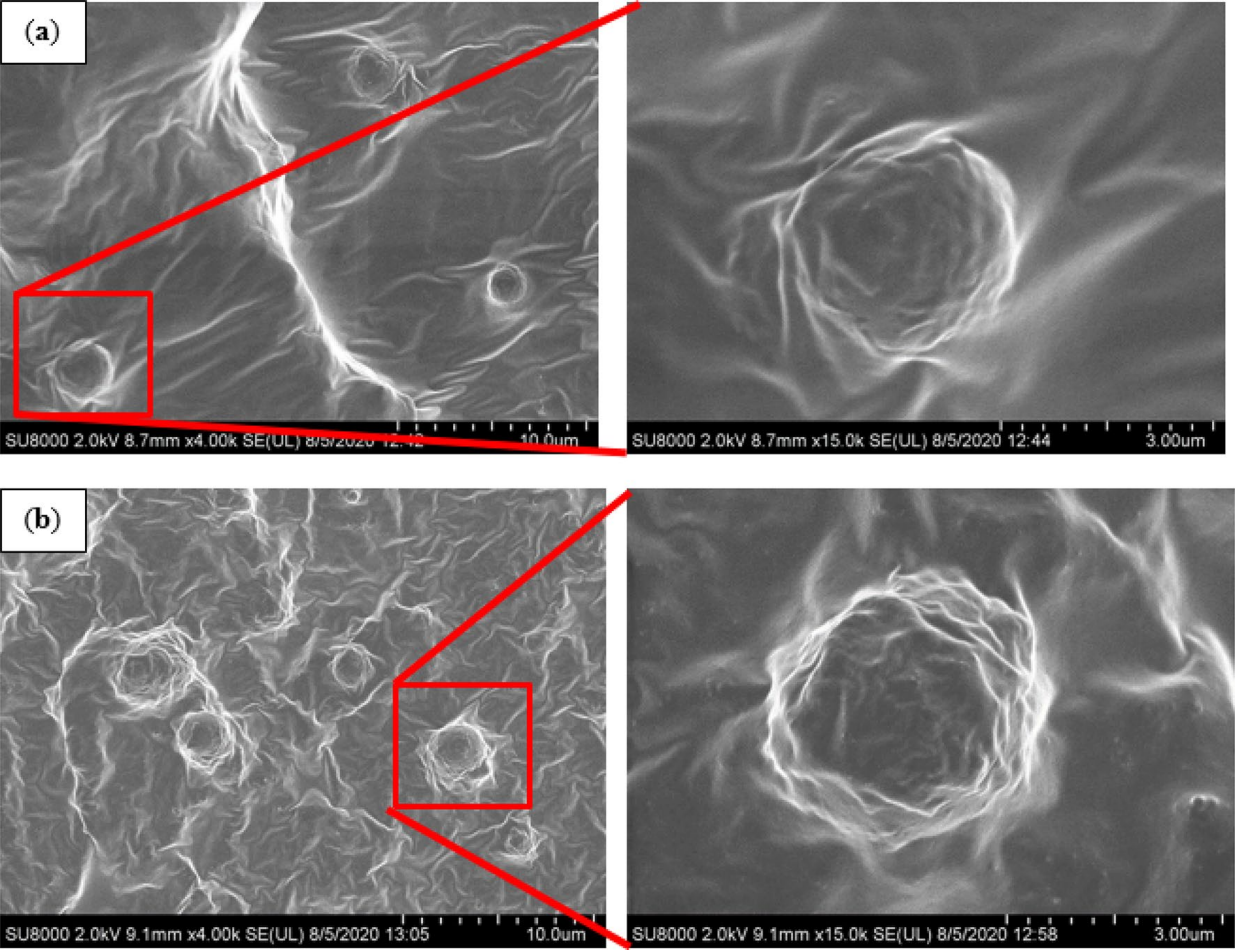
FESEM images of MRE containing embedded (**a**) non-corroded spherical CIPs and (**b**) corroded spherical CIPs (treated with 1.0 vol.% HCl).

Figure 6 shows the FESEM images of the MRE-S that was reinforced with non-corroded and corroded spherical CIPs (treated with 1.0 vol.% HCl). It can be observed in Fig. 6a that the surface of MRE with non-corroded spherical CIPs looked smooth and those particles were well-embedded within the rubber matrix, which suggests a good adhesion between the particles and the matrix phase. Meanwhile, in Fig. 6b, the image illustrates a rougher MRE-S surface as a result of embedded corroded CIPs into the rubber matrix. The corroded spherical CIPs were observed as being pulled out of the rubber matrix, showing a weak interaction between the particles and the rubber matrix due to incompatibility of the oxide layer existed in the MRE. Moreover, due to an extending contact under the acidic treatment. This kind of oxide layers might act as the ‘passive’ layers thus resulted in the discontinuity bonding of the particle–matrix interface bonding. The weak interaction between the particle–matrix interfaces occur that will weaken the efficiency of stress transfer from matrix to particles. When an external stress is applied onto the MRE, the stress transfer at the interface between the particles and matrix phases become weak. Therefore, it resulted the corrode CIPs seemed being pull out of the rubber matrix was observed. This finding is similar to the one reported by previous research^35^.

**Figure 7:**
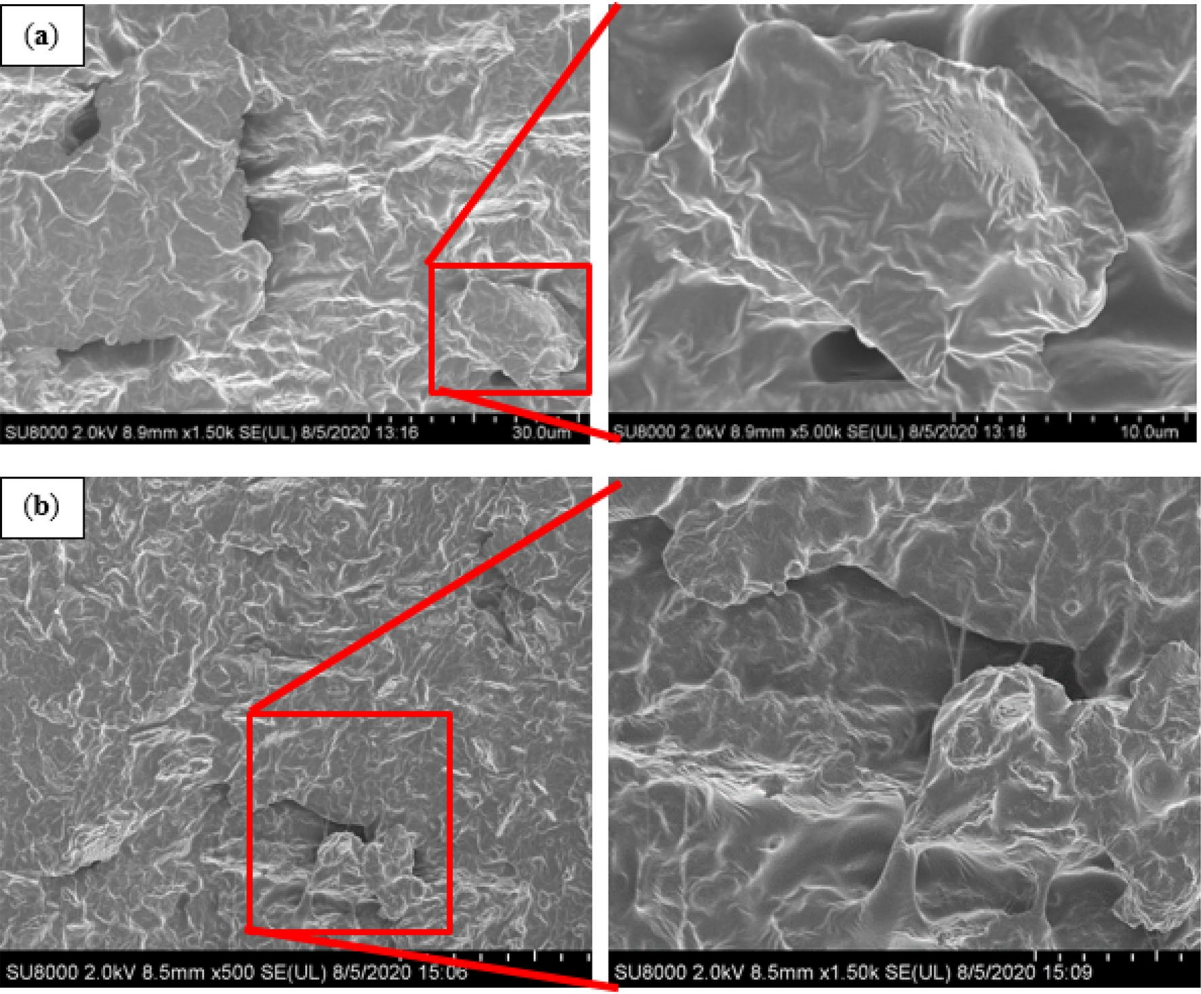
FESEM images of MRE containing embedded (**a**) non-corroded plate-like CIPs and (**b**) corroded plate-like CIPs (treated with 1.0 vol.% HCl).

The findings were further supported by FESEM images as presented in Fig. 7. In particular, the surface of the MRE with non-corroded plate-like CIPs was smoother, flattened, and those particles which embedded well in the rubber matrix, was believed to have a good interface bonding between the particles and the matrix (Fig. 7a). Meanwhile in Fig. 7b, the micrograph shows that the surface of MRE with embedded corroded plate-like CIPs were rougher. This might be due to a coarser topography of the corroded plate-like CIPs in the rubber matrix. In addition, the particle looked afloat and voided as a result of the oxide layer that led to the weak interaction between the particle and the matrix phase.

As stated earlier, the LVE region is important to determine the elastic limit of each sample at different conditions. Upon concluding the LVE region of the MRE samples, considering the types of MRE-S and MRE-P and in conditions of non-corroded and corroded CIPs, it was suggested that the LVE region for all samples was about 0.02% or 0.0002 of amplitude strain. This value was believed to cause an elastic behavior of the MRE upon experiencing applied stress to the material. The microstructure of MRE samples would remain unchanged which would appropriately undergo further analysis in other rheological tests.

**Figure 8:**
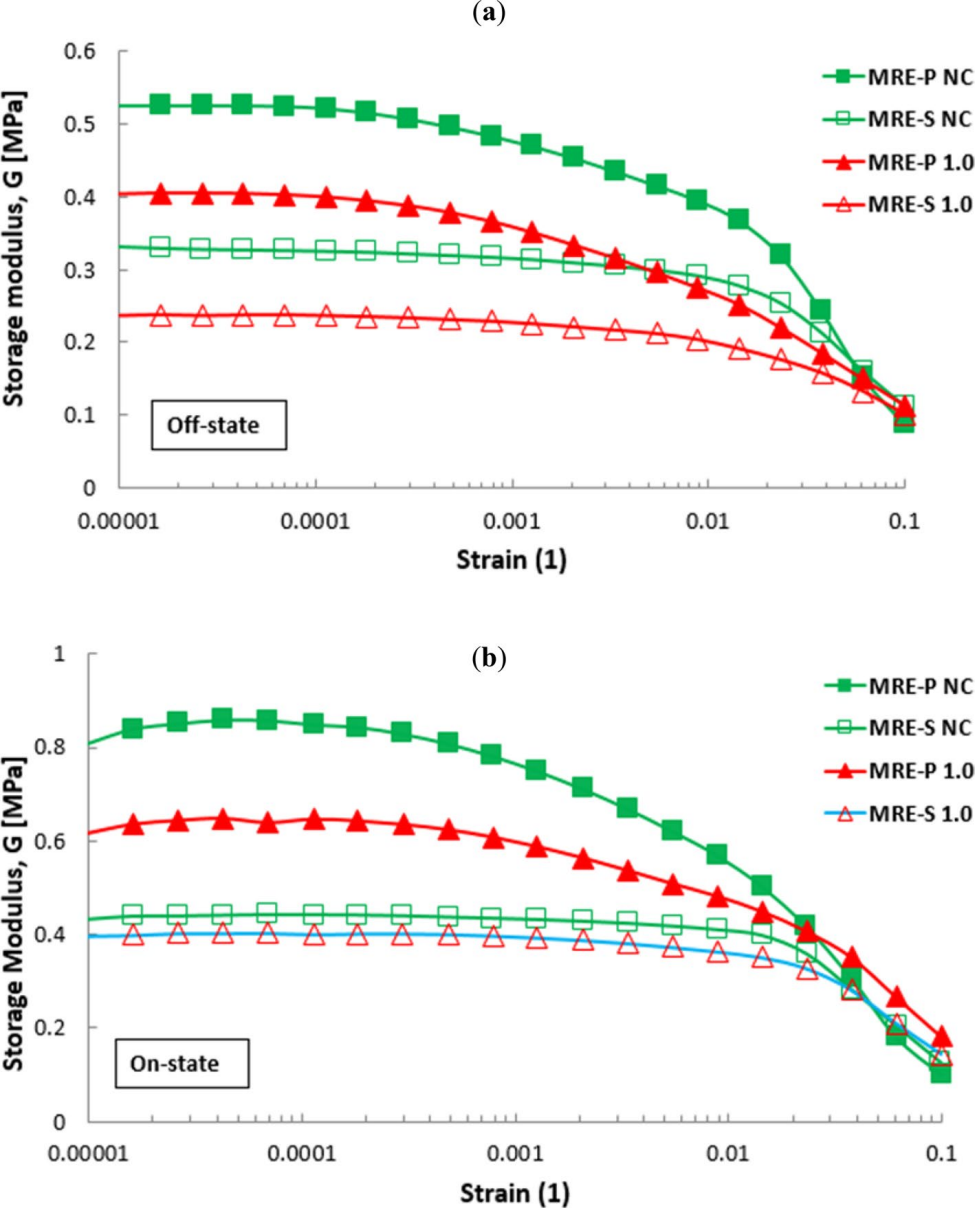
Comparison of the storage modulus of MRE samples containing spherical (unfilled symbol) and plate-like (filled symbol) CIPs, in non-corroded and corroded conditions towards the applied strains at (**a**) 0 Amps (off-state) and (**b**) 3 Amps (on-state).

Despite the previous graphs, Fig. 8 compares the results obtained from Figs. 4 and 5, particularly for MRE with non-corroded conditions of spherical and plate-like CIPs, and with both corroded CIPs for 1.0 vol.% HCl. The plotted graphs focus on the storage modulus of MRE samples in the absence and presence of the external magnetic field, respectively. As shown in Fig. 8a, briefly, there is a significant difference in terms of the storage modulus of MRE containing spherical and plate-like CIPs, either in non-corroded or corroded conditions. However, the MRE with plate-like CIPs exhibited a higher a storage modulus even with corroded conditions of CIPs at 1.0 vol.% HCl, compared to MRE with non-corroded circular CIPs. In this case, the shape of CIPs played a vital role in enhancing the performance MRE even in corroded condition.

Similarly, in Fig. 8b, the storage modulus of MREs with plate-like CIPs possessed a higher storage modulus. In fact, the value was greater for MRE with corroded plate-like CIPs in contrast to non-corroded spherical MRE, but still lower than that of MRE with non-corroded plate-like CIPs. Overall, the findings clearly showed that the storage modulus of MRE-P samples was greater as compared to MRE-S samples, even in corroded conditions. This was due to the larger surface area of plate-like CIPs which enhanced the particle–particle interactions in the matrix phase, especially at the on-state condition, thus increasing the capability to store more modulus. Nevertheless, all values of storage modulus of MRE samples at on-state condition (Fig. 8b) exhibited higher as a result of stiffer MRE that corresponded to the applied magnetic field. As the MRE with reinforced plate-like CIPs became stiffer, however, its oxide layer became more brittle and slightly lowered its LVE region. On the other hand, the presence of oxide layer on the CIPs surfaces, either in spherical or plate-like shapes, has caused a decrease in the storage modulus accordingly due to poorer interactions with to the applied magnetic field.

**Figure 9:**
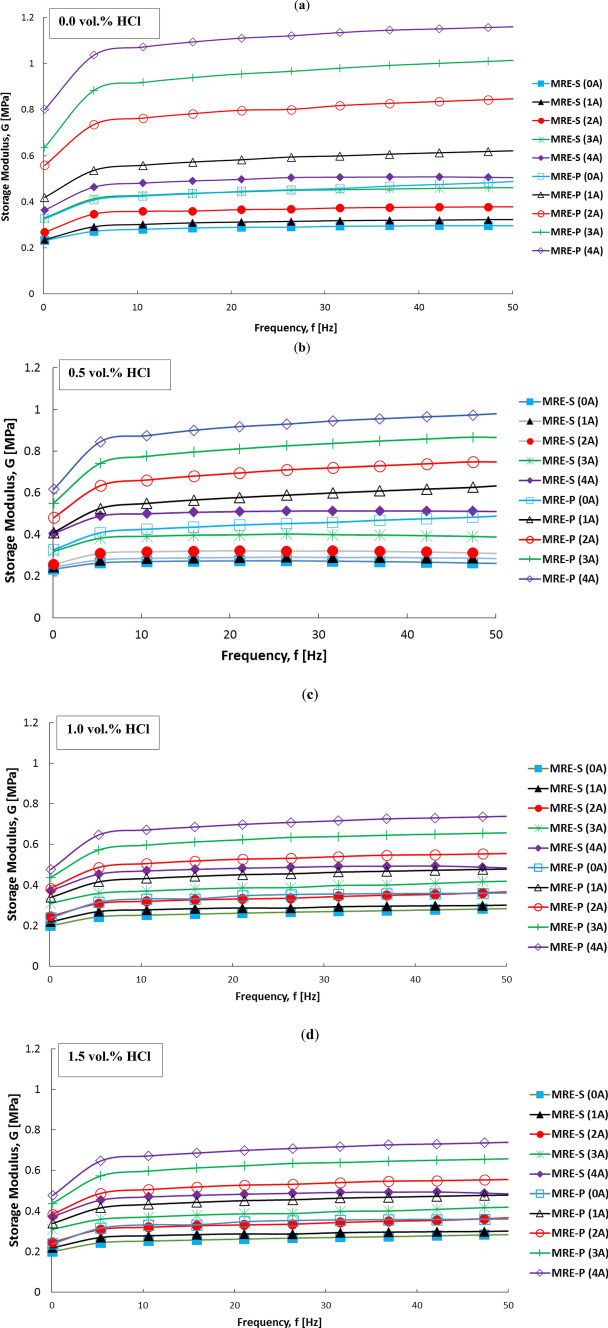
Variation of storage modulus of MRE samples towards frequency sweeps, containing spherical (filled symbol) and plate-like (unfilled symbol) CIPs, at different corrosion conditions of (**a**) 0, (**b**) 0.5, (**c**) 1.0 and (**d**) 1.5 vol.% HCl, under different applied magnetic fields.

The frequency test was carried out with the set strain amplitude of 0.02% and the frequency sweep in the range of 0.1–50 Hz. Figure 9 shows the storage modulus of MRE-P and MRE-S samples with different corroded rates of CIPs, respective to different concentrations of HCl with various magnetic fields as a function of frequency. The storage modulus of all MRE samples containing either non-corroded or corroded CIPs as observed in Fig. 9a–d increased with the increased of frequency at both conditions either with or without magnetic fields. In general, the results are consistent with previous findings^36,37^ as the phenomenon occurred could be attributed to the mismatching speed between the slower movement of polymer molecular chains and the rapid shear force applied to the sheet matrix^38^. The dynamic response time of the MRE decreased with the increasing frequency resulting from the failure of the polymer molecular chain of the matrix to complete stretching and shrinking over time. The increased frequency immobilized a tinier structure consisting of the elastomeric matrix and magnetic particles, which resulted in increased stiffness for the whole MRE system. The MRE samples experienced a linear increment in storage modulus for frequencies up to 7 Hz indicating a distinct stiffer of the MRE towards the applied frequencies and plateaued at higher frequencies. The higher frequency movements were relatively fast containing minimal relaxation process (time to take the polymer chains back to equilibrium after deformation), which were not very sensitive to structure of materials. Thus, while studying the effect of other factors in reducing the influence of frequency, it is more pronounced in the low-frequency range.

In addition, the initial values of the storage modulus of MRE-S and MRE-P samples were decreased, as the HCl concentrations increased, respectively. This result can be explained by the fact that the existence of the oxide layers on the surface of corroded CIPs, could weaken the interaction between the elastomeric matrix and magnetic particles and reduce the stiffness of the matrix. On the other hand, the storage modulus of MRE-P samples was noted to be higher than that of MRE-S samples, as a function of frequencies. Indeed, the trends showed increment with the increased of magnetic fields, representing the capability of MRE to store higher modulus or become stiffer with the excitation of magnetic fields. The differences in storage modulus between MRE-P and MRE-S samples are highlighted and summarized in Table 3. From this data, interestingly, it is apparent that the initial value of the storage modulus of the MRE-P samples was higher than that of the MRE-S samples for each different concentration of HCl. It is believed to occur due to the MRE with the reinforced plate-like shape of CIPs that had a larger surface area of the particle, the interference friction between the particles and the matrix became higher and hindered the particle sedimentation, resulting in a high stiffness. Therefore, this phenomenon was more pronounced when MRE samples were simultaneously put under higher magnetic fields.

**Table 3:** Initial and maximum storage modulus of MRE samples.

MRE samples	Initial storage modulus, G_o_					Maximum storage modulus, G_f_				
	Magnetic field (B)					Magnetic field (B)				
	0	1	2	3	4	0	1	2	3	4
MRE-S NC	0.232	0.236	0.269	0.331	0.364	0.262	0.298	0.361	0.447	0.505
MRE-S 0.5	0.230	0.243	0.257	0.319	0.405	0.182	0.198	0.200	0.293	0.461
MRE-S 1.0	0.224	0.239	0.258	0.316	0.381	0.332	0.375	0.429	0.480	0.518
MRE-S 1.5	0.200	0.218	0.245	0.311	0.374	0.314	0.327	0.385	0.432	0.512
MRE-P NC	0.327	0.419	0.558	0.636	0.801	0.573	0.762	0.902	1.051	1.183
MRE-P 0.5	0.327	0.409	0.541	0.551	0.717	0.571	0.721	0.821	1.040	1.114
MRE-P 1.0	0.317	0.408	0.512	0.546	0.691	0.409	0.668	0.809	0.946	1.084
MRE-P 1.5	0.239	0.340	0.384	0.436	0.477	0.339	0.488	0.571	0.684	0.756

**Figure 10:**
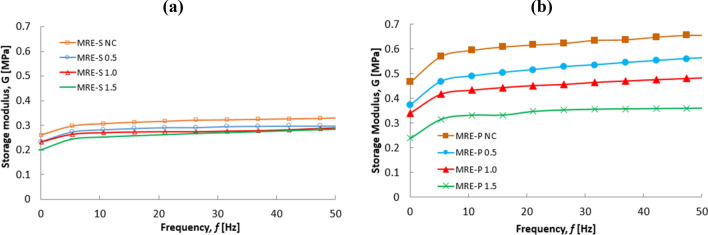
Storage modulus versus frequency of MREs containing (**a**) spherical and (**b**) plate-like shapes at off-state (0A) conditions.

The above results provide further support for the increment hypothesis on the storage modulus value of MRE-P samples. In the absence of applied magnetic field (off-state) condition, it should be noted that the storage modulus increased in parallel with the increment of frequency for non-corroded and corroded CIPs, either in a spherical shape (MRE-S) or plate-like shape (MRE-P) samples. However, it could be seen that the MRE-P samples exhibited a higher storage modulus compared to MRE-S samples. From the plotted graph in Fig. 10, it can be seen that the MRE-P samples were significantly affected by the performance of MRE in the absence of an applied magnetic field.

**Figure 11:**
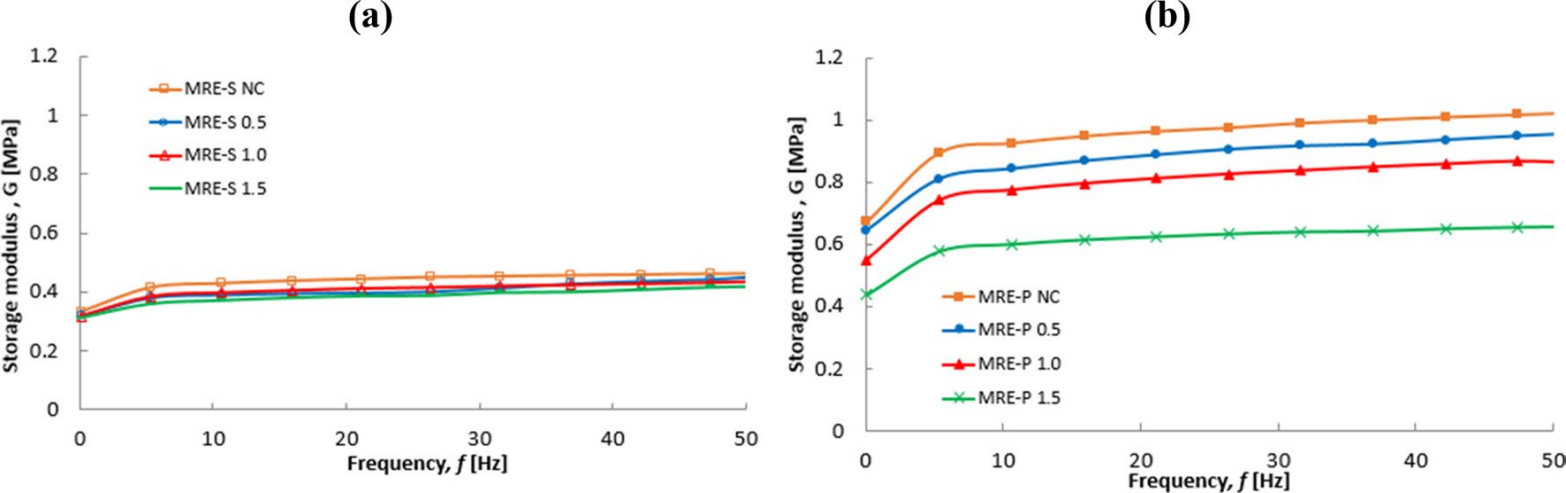
Storage modulus versus frequency of MREs containing different shapes, (**a**) spherical and (**b**) plate-like CIPs at 3A (on-state) condition.

The results of MREs under off- and on-state conditions were compared as shown in Fig. 10. The increment of initial storage modulus for all MRE samples was more pronounced in the presence of applied magnetic field (on-state). As shown in Fig. 10a, the MRE-S NC exhibited the highest initial storage modulus of 0.331 MPa as the frequency increased. After corrosion, the initial storage modulus of MRE-S containing corroded spherical CIPs, slightly decreased in the following order: MRE-S 0.5 > MRE-S 1.0 > MRE 1.5 with yield of 0.318, 0.316 to 0.310 MPa, respectively. It was noted that the storage modulus became smaller with a higher concentration of HCl. As seen in Fig. 10b, the MRE-P NC sample exhibited a higher initial storage modulus than other MRE-P samples, which was 0.672. Moreover, a similar trend was also observed for MRE-P samples, in which the MRE- P samples containing corroded CIPs exhibited a decrement trend for storage value. The increment of HCl content led to a lower initial storage modulus. As such, the lowest initial storage modulus of 0.337 MPa was documented for the MRE-P with corroded CIPs (1.5 vol.% HCl). Meanwhilet the MRE-P with 0.5 and 1.0 vol.% HCl corroded CIPs demonstrated the initial storage modulus of 0.551 and 0.489, respectively. Figure 11 shows the storage modulus of MRE-S and MRE-P samples as function of frequency at on-state (3A) condition.

As illustrated in Fig. 11a,b, the storage modulus for MRE-S and MRE-P samples with non-corroded CIPs exhibited the highest storage modulus. A similar trend for both samples of MRE- S and MRE-P containing corroded CIPs, the reduction of storage modulus is observed with an increase in HCl concentration. The significant decrease in storage modulus for corroded CIPs either in MRE-S or in MRE-P samples in Fig. 11 was due to the presence of thin layers on the surface of oxidized CIPs formed during the corrosion process. It is believed that the existence of these corrosive layers on the surface of plate-like CIPs had affected the performance of MRE accordingly. Likewise, it could be seen that the MRE-P samples exhibited a higher storage modulus compared to MRE-S samples in both conditions, off- and on-state.

**Figure 12:**
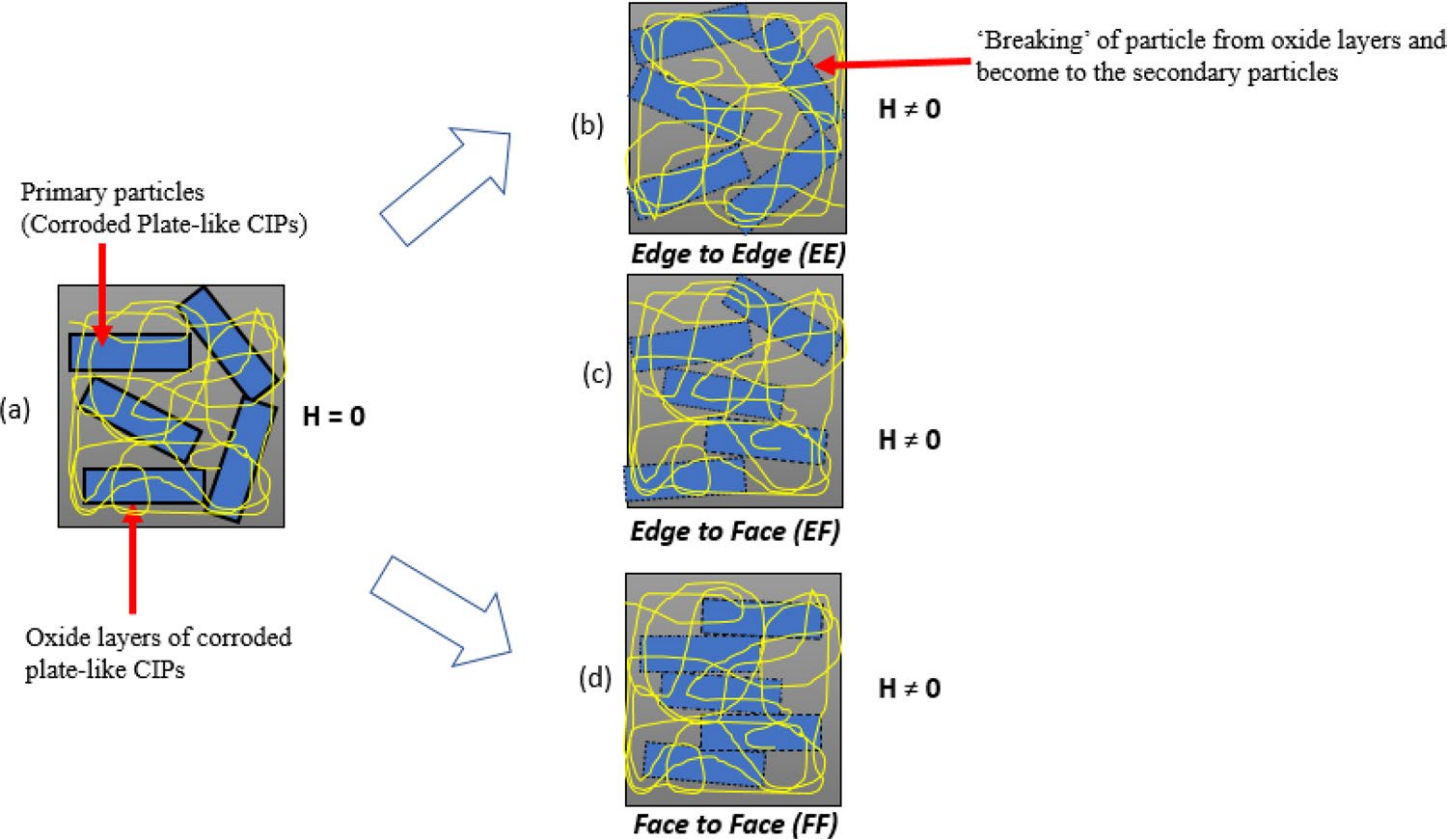
Schematic of the possible mechanisms of stacking interaction corroded plate-like CIPs in MRE-P under the applied magnetic field.

Moreover, as shown in Fig. 11b, the storage modulus of MRE-P was significantly higher, that is up to 1 MPa at a constant frequency of 50 Hz, compared to MRE-S samples in Fig. 11a at 0.4 MPa. The higher value of storage modulus for MRE-P samples indicated a good matrix-particle interaction and a stronger interparticle interaction between the plate-like particles inside the matrix. As the plate-like particles had a higher aspect ratio, it provided a larger contact area and easy magnetization axes leading to the reduction in the interparticle gap between the particles inside the matrix^25^. Therefore, in the presence of the external magnetic field (on-state), the interparticle interactions between the particles were improved as the particles became easier to magnetize and vibrated to form chains in the direction of the magnetic field^29,39^. Additionally, the higher storage modulus of MRE-P samples was represented by the increment of their material stiffness even though they contained the corroded plate-like particles. A possible mechanism for MRE-P samples with the corroded plate-like interaction is shown in Fig. 12. Thus far, the evidence supports the idea on how the storage modulus of MRE-P samples containing corroded particles was improved under the presence of the magnetic field.

In the presence of a magnetic field, when the applied load is transmitted through the matrix to the embedded corroded plate-like CIPs by shear stress, the high interparticle chain stress might occur and leads to the surface of the oxide layer. The chain may fracture and break into small pieces, and separate from the primary particles and act as the secondary particles due to the ‘breaking’ of particles. This ‘breaking’ phenomenon could lead to a slightly reduced interparticle gap among them, by enhancing the ability to store elastic energy due to good stacking interaction. This is because the chain structures of magnetically plate-like CIPs are stronger due to possible distinct types using a stacking mode interaction. As shown in Fig. 12, after the magnetic field was externally is applied, three different stacking modes of particle association might happen; the stacking mode is called edge to edge (EE) as shown in Fig. 12b. A Fig. 12c indicates another possibility of stacking mode in edge to face (EF) association. In addition, the interaction of the stacking modes could be due to face to face (FF) association in Fig. 12d. The combination of phenomenon is almost similar to the one reported by^40^. These possible stacking mode interactions decreased of interparticle gap corroded plate-like is assumed to produce better stacking interaction in the presence of a magnetic field, resulting in higher storage modulus compared to MRE-S samples with corroded spherical particles.

### Current sweep measurement

Relative MR effect is related to the performance of MRE-S and MRE-P samples under an oscillatory shear mode. For evaluation of the MR effect of MRE, the following equation was referred to such that:

1 $$ {\text{Relative MR effect}} = \frac{{\left( {G_{\max } - G_{0} } \right)}}{{G_{0} }} \times 100\% $$

where *G*_0_ is the initial storage modulus, and *G*_*max*_ is the maximum storage modulus at the highest magnetic flux density^41,42^.

**Figure 13:**
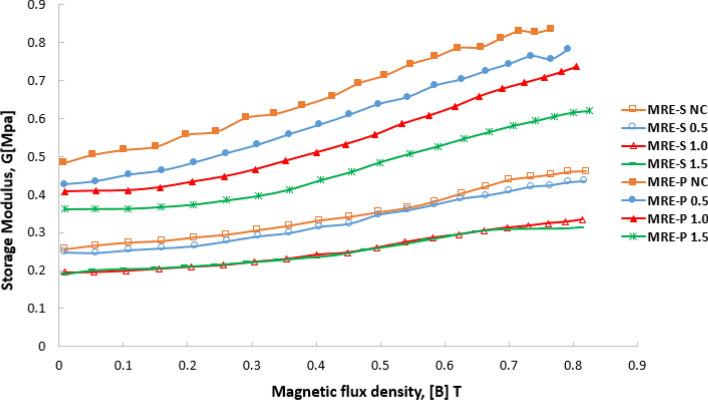
Change of stiffness or storage modulus corresponding to various magnetic flux densities, B for MRE samples containing different corrosion conditions of spherical and plate-like CIPs.

Figure 13 presents the dependence of the storage modulus of MRE-S (empty points) and MRE-P (filled points) samples at various magnetic flux densities, B at a constant strain amplitude and the frequency at 0.02% and 1 Hz, respectively. As the magnetic flux density increased, the storage modulus for all MRE-S samples increased, indicating a further stiffer material. In contrast, for MRE-P samples, when the magnetic flux density was small (< 0.2 T), the storage modulus increased slowly, and then, rapidly increased as the external magnetic field increased. On top of that, the storage modulus of the MRE-P samples was larger compared to the MRE-S samples for each corrosion condition. It is believed to be due to the stronger magnetic interaction between the adjacent plate-like particles along the field direction, causing stiffer or higher storage modulus for all MRE-P samples.

To quantitatively describe the MR effect of the MRE samples at various magnetic field strengths, the absolute and relative MR effect values are summarized in Table 4. It should be noted that the MRE-P samples exhibited a higher initial storage modulus due to a stiffer material at the initial state. Even though the saturation magnetization, M_s_ of MRE-S samples was larger than that of MRE-P, however, a higher storage modulus possessed by MRE-P samples caused relative and absolute MR effect which were enhanced up to 90% as compared to MRE-S samples with around 80%.

**Table 4:** Absolute and relative MR effect for all MREs.

Concentration of HCl (%)	Initial modulus, *G*_*o*_ (MPa)	Absolute MR effect, *∆G* (MPa)	Relative MR effect [%]
MRE-S NC	0.255	0.206	80.8
MRE-S 0.5	0.248	0.187	75.5
MRE-S 1.0	0.195	0.138	70.7
MRE-S 1.5	0.190	0.122	64.2
MRE-P NC	0.482	0.353	90.6
MRE-P 0.5	0.420	0.361	87.0
MRE-P 1.0	0.407	0.329	81.8
MRE-P 1.5	0.361	0.258	71.7

At a stacking mode in MRE, it is believed that the larger area of plate-like CIPs was closer to one another within the elastomeric matrix. This condition led to a stronger dipole–dipole interaction between the neighboring particles when the MRE-P samples were subjected to an external magnetic field, resulting in stiffness enhancement. In contrast, for MRE-P samples containing corroded CIPs, the observed decrement in the storage modulus and MR effect of MRE could be attributed to the existence of the oxide layer. Thus, the response of MRE towards the applied magnetic field had reduced and the value was further decreased as more corrosion effects were experienced by the CIPs. However, the values of storage modulus of MRE-P were still larger as compared to MRE-S samples although at the corroded CIPs condition, due to closer plate-like CIPs to one another caused a slightly stronger magnetic moment towards the magnetic field.

## Conclusions

In this investigation, rheological tests have been performed on the MREs containing non-corroded and corroded CIPs in order to clarify the influence of different shapes of corroded CIPs on the rheological properties of MRE. The results have been drawn as follows;

- Significance changes in the morphology of corroded CIPs after immersion in a 1.0 vol% of HCl exhibited the existence of a corrosive layer known as the fluffy ice crystal-like structure on the outer surface of the CIPs for the spherical of corroded CIPs. In contrast, the white dots were observed on the outer surface of corroded plate-like CIPs.
- The results showed that the saturation magnetization of plate-like CIPs was lower than spherical CIPs, and the corroded CIPs for both shapes decreased with the increase of HCl concentration.
- The rheological properties demonstrated an enhancement up to 90% of the MR effect of the MRE-P compared to the MRE-S, which showed that stronger interparticle interactions were formed due to larger surface area owing to the shape of plate-like CIPs. However, the increasing gap interaction between particles due to the existence of corrosive layers during corrosion of embedded CIPs resulted in a decrement of MR effect. These findings directly indicate that the particle shape of CIPs can alter the rheological properties in MRE performance.

Therefore, the results presented in this work have laid an important platform for investigating how different shapes of CIPs can be used in prolonging the MRE’s performance.
